# ROS-p53-cyclophilin-D signaling mediates salinomycin-induced glioma cell necrosis

**DOI:** 10.1186/s13046-015-0174-1

**Published:** 2015-05-30

**Authors:** Li-sen Qin, Pi-feng Jia, Zhi-qing Zhang, Shi-ming Zhang

**Affiliations:** Department of Neurosurgery, the First Affiliated Hospital of Soochow University, No. 188, Shi-zi Street, Suzhou, Jiangsu People’s Republic of China; Department of Neurosurgery, the Sixth People’s Hospital of Yancheng, Yan-cheng, Jiangsu People’s Republic of China; Department of Neurosurgery, Shanghai Ruijin Hospital North, School of Medicine, Shanghai Jiao Tong University, Shanghai, People’s Republic of China; Jiangsu Key Laboratory of Translational Research and Therapy for Neuro-Psycho-Diseases and Institute of Neuroscience, Soochow University, Suzhou, Jiangsu 215021 China

**Keywords:** Salinomycin, Glioma, Programmed necrosis, Mitochondrial permeability transition pore (mPTP), Cyclophilin-D and p53

## Abstract

**Background:**

The primary glioblastoma multiforme (GBM) is the most malignant form of astrocytic tumor with an average survival of approximately 12–14 months. The search for novel and more efficient chemo-agents against this disease is urgent. Salinomycin induces broad anti-cancer effects; however, its role in GBM and the underlying mechanism are not clear.

**Results:**

Here we found that salinomycin induced both apoptosis and necrosis in cultured glioma cells, and necrosis played a major role in contributing salinomycin’s cytotoxicity. Salinomycin induced p53 translocation to mitochondria, where it formed a complex with cyclophilin-D (CyPD). This complexation was required for mitochondrial permeability transition pore (mPTP) opening and subsequent programmed necrosis. Blockade of Cyp-D by siRNA-mediated depletion or pharmacological inhibitors (cyclosporin A and sanglifehrin A) significantly suppressed salinomycin-induced glioma cell necrosis. Meanwhile, p53 stable knockdown alleviated salinomycin-induced necrosis in glioma cells. Reactive oxygen species (ROS) production was required for salinomycin-induced p53 mitochondrial translocation, mPTP opening and necrosis, and anti-oxidants n-acetylcysteine (NAC) and pyrrolidine dithiocarbamate (PDTC) inhibited p53 translocation, mPTP opening and glioma cell death.

**Conclusions:**

Thus, salinomycin mainly induces programmed necrosis in cultured glioma cells.

**Electronic supplementary material:**

The online version of this article (doi:10.1186/s13046-015-0174-1) contains supplementary material, which is available to authorized users.

## Background

The primary glioblastoma multiforme (GBM) is the most malignant form of astrocytic tumor, which has one of the worst prognoses among all human tumors, with a median survival of approximately 12 months [1–7]. In the past three decades, postoperative radiation and/or temozolomide (TMZ) become the current standard care for GBM [1, 3, 4]. However, there has been no significant improvement in the overall survival [1, 3, 4]. One key hurdle is the molecular heterogeneity of GBM, which impedes uniform application of specific molecularly targeted agents [2]. Thus, the search for novel and more efficient chemo-agents against this deadly disease is urgent and important [2].

The results of a recent high-throughput screening study indentified that salinomycin, an antibacterial and coccidiostat ionophore therapeutic drug, selectively kills cancer stem cells from tumorspheres, and inhibits tumor growth in mice [8]. Since then, a number of groups have studied salinomycin as a potential anti-cancer agent [8, 9], and results have shown that salinomycin inhibits the growth of various immortalized cancer cells both *in vivo* and *in vitro* [10–13]. However, the underlying mechanisms are not fully understood, although Wnt suppression [11], p-glycoprotein inhibition [9] and reactive oxygen species (ROS) production [12] have been associated with salinomycin-mediated anti-cancer effects. In the current study, we investigated the potential role of salinomycin in glioma cells, and studied the molecular mechanisms involved.

It has been long believed that necrotic cell death is a passive and uncontrolled form of cell death. Recently, however, it is discovered that necrosis, similar to apoptosis, is also a molecularly regulated event that is happening in a number of stress conditions [14–19]. Further studies have found that mitochondrial permeability transition pore (mPTP), the mitochondrial channel complex, plays a vital role in mediating this “programmed necrosis” [17–20]. MPTP is composed of at least three primary components, including the voltage-dependent anion channel (VDAC), the adenine nucleotide translocator-1 (ANT-1) and the mitochondrial matrix protein cyclophilin D (Cyp-D) [17, 20, 21]. Cyp-D is known to sit in the mitochondrial matrix to keep the mPTP closed [20–22]. Under stress conditions, i.e. Ca^2+^ [14, 23], hypoxia [14, 23], ROS [24], UV radiation [25], Cyp-D will associate with ANT-1 in the inner membrane, open the mPTP pore, cause mitochondrial membrane potential (MMP) loss, mitochondria swelling, Ca^2+^ release, ROS production, and eventually leading to cell necrosis. Interestingly, recent studies have implicated the important role of Cyp-D dependent mPTP opening in certain chemo-drugs-induced cancer cell necrosis [26, 27]. In the current study, we found that salinomycin induced programmed necrosis in cultured glioma cells.

## Methods

### Chemical and reagents

Salinomycin, sanglifehrin A (SfA), cyclosporine A (CsA), n-acetyl cysteine (NAC), temozolomide (TMZ) and pyrrolidinedithiocarbamate (PDTC) were purchased from Sigma (St. Louis, MO). Necrostatin-1 (Nec-1) was purchased from Cayman Chemical (Beijing, China). Antibodies against tubulin and Cyp-D were purchased from Santa Cruz Biotech (Santa Cruz, CA), antibodies for p53 (regular and specific sites of phosphorylation) were purchased from Cell Signaling Technology (Danvers, MA).

### Cell culture

U87MG, U251MG and EFC-2 glioma cells were maintained in dulbecco’s modified Eagle’s medium (DMEM, Sigma, St. Louis, MO), supplemented with a 10 % fetal bovine serum (FBS, Sigma), penicillin/streptomycin (1:100; Sigma) and in a CO_2_ incubator at 37 °C.

### Primary culture of mouse astrocytes

Tissues from whole brains of post-natal (P1–P2) mice were triturated, and then cells were placed on poly-d-lysine pre-coated cell culture flasks in DMEM containing 15 % FBS, 100 U/ml penicillin, and 100 μg/ml streptomycin. Cultures were maintained at 37 °C in a humidified atmosphere of 5 % CO_2_/95 % filtered air. After reaching a confluent monolayer of glial cells (10–14 days), microglia were separated from astrocytes by shaking off for 5 h at 100 rpm. The enriched astrocytes were >96 % positive for glial fibrillary acidic protein (GFAP).

### Cell viability MTT assay

The cell viability was measured by the 3-[4,5-dimethylthylthiazol-2-yl]-2,5 diphenyltetrazolium bromide (MTT) (Sigma, St. Louis, MO) method as reported [28]. Briefly, cells were seeded in 96-well plates with 70–80 % confluence. After indicated treatment/s, MTT tetrazolium salt (0.25 mg/ml) was added to each well for 2 h at 37 °C. Afterwards, 200 μl of DMSO was added to dissolve formazan crystals. The absorbance of each well was observed by a plate reader at a test wavelength of 490 nm. The value of each treatment group was expressed as percentage change of that of control group.

### “Dead” cell detection by trypan blue staining

As reported [28], the number of dead glioma cells (trypan blue positive) after treatment was recorded, and the percentage (%) of dead cells was calculated by the number of the trypan blue stained cells divided by the total cell number, which was automatically tested by a handheld automated cell counter (Merck Millipore, Shanghai, China).

### Clonogenicity assay

As reported [28], U87MG cells (5× 10^3^) were suspended in 1 ml of DMEM containing 0.1 % agar (Sigma, St. Louis, MO), 10 % FBS and with indicated treatments or the vehicle control. The cell suspension was then added on top of a pre-solidified 100 mm culture dish. The medium was replaced every two days. After 10 days of incubation, colonies were photographed at 4×. The number of large colonies (>50 μm in diameter) was manually counted and recorded, and the number was expressed as percentage change of the control group.

### Analysis cell apoptosis and necrosis by fluorescence-activated cell sorting (FACS) sorting propidium iodide (PI) -Annexin V staining

As reported [28], the glioma cell apoptosis and necrosis were determined by the Annexin V *In Situ* Cell Apoptosis Detection Kit (Roche Molecular Biochemicals, Indianapolis, IN, USA) according to the manufacturer’s protocols. Briefly, after indicated treatment/s, glioma cells were stained with Annexin V and propidium iodide (PI) (Molecular Probes). The cell apoptosis percentage was reflected by Annexin V^+/+^/PI^−/−^ plus Annexin V^+/+^/PI^+/+^ percentage detected by fluorescence-activated cell sorting (FACS) (BD, Shanghai, China). Annexin V^−/−^/PI^+/+^ percentage was utilized as an indicator of cell necrosis. The time point for FACS assay was selected based on pre-experiment results.

### Caspase-3 activity assay

The cytosol proteins of approximately 2 × 10^6^ glioma cells were extracted in cell lysis buffer containing 25 mm HEPES, 5 mm MgCl_2_, 5 mm EDTA, 5 mm dithiothreitol and 0.05 % phenylmethylsulfonyl fluoride, (pH 7.5). Twenty μg of cytosolic extracts were added to caspase assay buffer (312.5 mM HEPES, pH 7.5, 31.25 % sucrose, 0.3125 % CHAPS) with benzyloxycarbonyl-DEVD-7-amido-4-(trifluoromethyl)-coumarin as substrate (Calbiochem, Darmstadt, Germany). After 2 h of incubation at 37 °C, the release of 7-amido-4-(trifluoromethyl) coumarin (AFC) was quantified, using a Fluoroskan system set to an excitation value of 355 nm and emission value of 525 nm. The value of each result was normalized, and was expressed as fold change vs. that of vehicle control group.

### Protein isolation and Western blots

Cells were washed with ice-cold PBS and then lysed using lysis buffer containing 1 % Nonidet P-40, 1 % deoxycholate, 0.1 % sodium dodecyl sulfate, 150 mmol/L sodium chloride and 10 mmol/L Tris–HCl (pH, 7.4). The lysates were collected and centrifuged. The concentration of the extracted protein was measured by bicinchoninic acid assay kit (Sigma). The extracted protein was boiled for 5 min in loading buffer. Samples were separated on 10 % SDS-polycrylamide gel, and after electro-blotting onto polyvinylidene fluoride (PVDF) membranes (Millipore, Shanghai, China), the membranes were blocked with blocking solution [10 % (w/v) milk in Tris-buffered solution plus Tween-20 (TBST), incubated overnight at 4 °C with primary antibodies, and then incubated with HRP-conjugated anti-rabbit/mouse second antibodies. The detection was performed by Super-signal West Pico Enhanced Chemiluminescent (ECL) Substrate according to the manufacturer’s protocol. The blot intensity was quantified and normalized as previously reported [28]. For detecting proteins in the mitochondria, intact mitochondria of cultured 2.0 × 10^7^ glioma cells were isolated using the “Mitochondria Isolation Kit for Cultured Cells” (Pierce, Rockford, IL).

### Immuno-precipitation (IP)

After treatment, 600 μg of cell lysates from mitochondrial fractions of glioma cells were pre-cleared. The supernatant was then rotated overnight with 2 μg of anti-Cyp-D (Santa Cruz Biotech). Next, the lysates were centrifuged for 5 min at 4 °C in a micro-centrifuge to remove nonspecific aggregates. The protein A/G PLUS-agarose (35 μl, Sigma) was then added to the supernatants for 4 h at 4 °C. Pellets were washed six times with PBS, resuspended in lysis buffer, and then assayed by Western blots. The time point for IP was based on previous publications and results from pre-experiments.

### Cyp-D siRNA

The SiRNA duplexes against human cyclophilin-D were purchased from Santa Cruz. Lipofectamine^TM^ 2000 was applied to transfect RNAi (100 nM) into cultured glioma cells according to manufacturer’s protocol. The same amount of scramble non-sense siRNA (control siRNA, Santa Cruz) was transfected into control cells. After 48 h, the Cyp-D expression and the equal loading in transfected cells were examined by Western blots. Only successfully transfected cells, demonstrated by markedly reduced level of Cyp-D, were used for further experiments. The transfection was repeated one more round if necessary.

### Detection of mitochondrial membrane potential (MMP)

Similar to previous reported, the MMP of glioma cells was measured through JC-10 dye (Invitrogen, Carlsbad, CA) [29]. The JC-10 dye exhibits two staining spectra. In normally resting cells, the dye forms aggregates in the mitochondrial membrane, exhibiting orange fluorescence. When the membrane potential is decreasing, the monomeric JC-10 will form in the cytosol, exhibiting the green fluorescence. Thus, the intensity of green fluorescence can be used as indicator of MMP loss. Briefly, after treatment, glioma cells were stained with 5.0 μg/ml of JC-10 for 5 min at room temperature under dark. Cells were then washed twice with warm PBS, and resuspended in fresh culture medium and read immediately on a microplate reader with an excitation filter of 485 nm. The OD value was used as an indicator of MMP loss.

### Cyp-D vector and transfection

The wt-Cyp-D plasmid (pSuper-puromycin-GFP- Cyp-D) and the empty vector (pSuper-puromycin) were gifts from Dr. Wang [29]. Lipofectamine^TM^ and PLUS reagent (Invitrogen, Carlsbad, CA) were applied to transfect Cyp-D plasmid or the vector (1 μg/ml) into cells according the manufacturer’s protocol. The stable clones were selected by puromycin (0.25 μg/ml medium). The puromycin-containing medium was refreshed every 3 days, until single resistant colony can be formed (4 weeks). The Cyp-D expression in the stable clones was always detected by Western blots to confirm the transfection efficiency. Only colonies with over-expressed Cyp-D were utilized for experiments.

### P53 shRNA knockdown and stable cell selection

P53 shRNA containing lentiviral particles were added to cultured glioma cells at the dose of 20 μl/ml medium, the infection took 48 h, afterwards, puromycin (0.25 μg/ml) was added to select the stable clones. The puromycin-containing medium was refreshed every 3 days, until single resistant colony can be formed (4 weeks). P53 expression in the stable colony was always detected by Western blots to confirm the infection efficiency. Control cells were infected with same concentration of scramble shRNA containing lentiviral particles.

### Reactive oxygen species (ROS) detection

Intracellular ROS generation was measured by flow cytometry using dichlorofluorescin (DCF) oxidation assay [30]. DCFH-DA enters passively into cells and is cleaved by nonspecific cellular esterase and oxidized in the presence of ROS. Briefly, 3*10^5^ glioma cells were plated in 60-mm culture plates overnight. After treatment, cells were incubated with DCFH-DA (5 μM) for 1 h at 37 °C. Thereafter, cells were washed with PBS and kept in 1 ml PBS, ROS fluorescence was analyzed using flow cytometer. The value of treatment group was expressed as fold change to that of untreated control group.

### The superoxide dismutase (SOD) activity assay

The SOD activity was measured using a SOD activity assay kit (BioVision, Mountain View, CA) according to the manufacturer’s protocol. Briefly, cells were seeded onto 100-mm^2^ dishes at a density of 5 × 10^6^ cells/dish. After treatment, the cells were washed with PBS, scraped from the plates into 1 mL of ice-cold PBS (containing 0.05 mM of EDTA), and homogenized. The homogenate was centrifuged at 4000 × g for 30 min at 4 °C. The resulting supernatant (20 μl) was added with 200 μl of water soluble tetrazolium working solution and 20 μL of enzyme working solution to a 96-well plate. After incubating the plate at 37 °C for 20 min, the absorbance at 450 nm was read using a microplate reader.

### Xenograft assays

Nude mice were purchased from Suzhou University Institute of Biological Science. U251 cells (10^6^ cells in 100 μL of saline/Matrigel (BD Pharmigen San Jose, Ca), 1:1 v/v) were injected subcutaneously into the right flank of 4-weeks-old female mice. Treatments were started after tumor reached approximately 200 mm^3^ (around 4 weeks after inoculation). Salinomycin (5.0 mg/kg) or with CsA (5.0 mg/kg) were administered to mice (10 per group) once daily for two weeks, intraperitoneally. The size of the tumors was measured by caliper every week, and tumor volumes were calculated using the following formula: π/6 × width ^2^× length. Control mice received vehicle only with the same schedule. All animal procedures were performed according to the Animal Experimentation guidelines upon approval of the experimental protocol by Soochow University and Shanghai Jiao Tong University. The study was approved by Soochow University and Shanghai Jiao Tong University review boards. All investigations were conducted according to the principles expressed in the Declaration of Helsinki.

### Statistical analysis

The data presented in this study were means ± standard deviation (SD). Statistical differences were analyzed by one-way *ANOVA* followed by multiple comparisons performed with post hoc Bonferroni test (SPSS version 18). Values of *p* < 0.05 were considered statistically significant. The difference between two groups was tested using paired-samples *t* test.

## Results

### Salinomycin inhibits proliferation and survival of cultured glioma cells *in vitro*

**Fig. 1:**
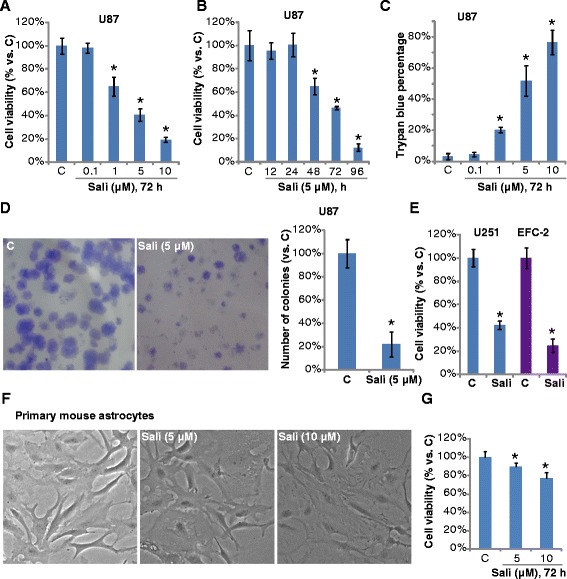
Salinomycin inhibits proliferation and survival of cultured glioma cells *in vitro*. U87MG glioma cells were treated with indicated concentration of salinomycin (Sali) for 72 h, or treated with 5 μM of salinomycin (Sali) for indicated time, cell viability and cell death were analyzed by MTT assay (**a** and **b**) and trypan blue staining (**c**) respectively. U87MG cells were cultured in salinomycin (Sali, 5 μM) containing medium for 10 days, and the left survival colonies were counted, representative images were also shown (**d**). U251MG and EFC-2 glioma cells were treated with salinomycin (Sali, 5 μM) for 72 h; cell viability was tested by MTT assay (**e**). Primary mouse astrocytes were treated with indicated concentration of salinomycin (Sali) for 72 h, cell morphology and viability were shown in (**f**) and (**g**). Experiments were repeated four times in this figure, and similar results were obtained. Error bars indicate standard deviation (SD). *p < 0.05 vs. untreated control (“c”) group. Magnification 1:400 for (**f**)

We first tested the potential role of salinomycin in cultured glioma cells. MTT cell viability assay results in Fig. 1a demonstrated that salinomycin dose-dependently inhibited U87MG cell survival. Meanwhile, the anti-survival effect of salinomycin was time-dependent (Fig. 1b). The viability of U87MG cells started to decrease 48 h after salinomycin (5 μM) treatment in U87MG cells (Fig. 1b). Using the trypan blue staining assay, we found that salinomycin treatment caused significant cytotoxicity of U87MG cell death, and the effect again was dose-dependently (Fig. 1c). To further confirm the potential role of salinomycin on proliferation of glioma cells, clonogenicity assay was performed, and results showed that salinomycin (5 μM) significantly reduced the number of survival colonies of U87MG cells (Fig. 1d). Figure 1e demonstrated that salinomycin inhibited the viability of U251MG and EFC-2 glioma cells. We also tested the potential cytotoxicity of salinomycin against primary cultured mouse astrocytes, and found that salinomycin (5–10 μM) only slightly inhibited the survival of primary astrocytes (Fig. 1f and g).

Together, these results show that salinomycin inhibits proliferation and survival of cultured glioma cells *in vitro*.

### Salinomycin mainly induces necrotic death of glioma cells

**Fig. 2:**
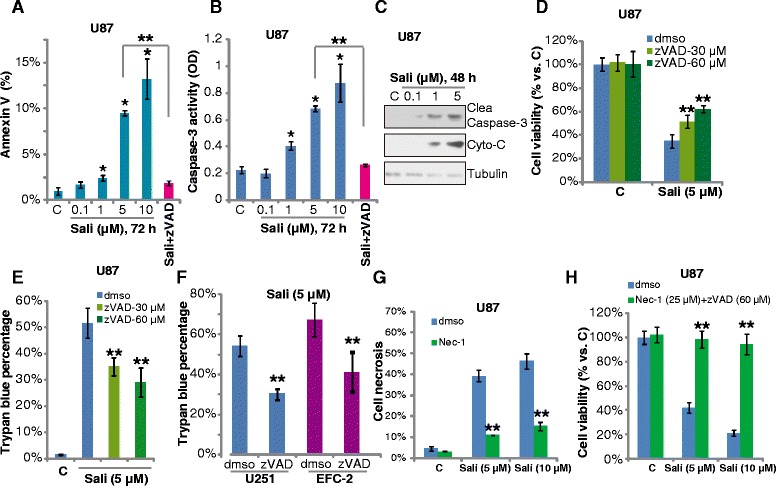
Salinomycin induces both apoptotic and necrotic death of glioma cells. U87MG cells were treated with indicated concentration of salinomycin (Sali) for 72 h, cell apoptosis was analyzed by Annexin V FACS assay (**a**) and caspase-3 activity assay (**b**). The effect of zVADfmk (60 μM) on salinomycin (Sali, 5 μM)-induced apoptosis was also shown (**a**-**b**). The expression of cleaved-caspase-3, cytochrome C and tubulin in cytosol after indicated salinomycin (Sali) stimulation was tested by Western blotting (**c**). U87MG cells were pre-treated with an apoptosis inhibitor zVADfmk (30 or 60 μM) for 1 h, followed by salinomycin (Sali, 5 μM) stimulation, cells were further cultured, MTT assay (**d**, 72 h) and trypan blue staining (**e**, 72 h) were performed. U251MG and EFC-2 glioma cells were treated with salinomycin (Sali, 5 μM) in the presence or absence of zVADfmk (zVAD, 60 μM) for 72 h, cell death was tested by trypan blue staining assay (**f**). U87MG cells were pre-treated with a necrosis inhibitor Nec-1 (25 μM) for 1 h, followed by salinomycin (Sali, 5/10 μM) stimulation, cells were further cultured for 72 h, cell necrosis was analyzed by FACS (**g**). The cell viability of U87MG cells with indicated treatment was tested (**h**). Experiments were repeated four times in this figure, and similar results were obtained. Error bars indicate SD. *p < 0.05 vs. Ctrl group (**a-b**). **p < 0.05 vs. Sali group (**d-g**)

The results above confirmed the cytotoxic effect of salinomycin in glioma cells; we then explored the role of apoptosis and necrosis in it. By using two different assays including Annexin V-FACS assay and caspase-3 activity assay, our results demonstrated that salinomycin induced moderate cell apoptosis (less than 15 %) in U87MG cells (Fig. 2a and b), which was blocked by the apoptosis inhibitor zVADfmk (Fig. 2a and b). Meanwhile, the Western blot results in Fig. 2c showed caspase-3 cleavage and cytochrome C release in salinomycin-treated U87MG cells, further supporting apoptosis induction. Note that less than 10 % cells were apoptotic after 5 μM of salinomycin stimulation in U87MG cells (Fig. 2a). Further, zVADfmk only slightly inhibited salinomycin-induced viability reduction and cell death in cultured glioma cells (Fig. 2d-f). On the other hand, we noticed a large proportion of necrotic U87MG cells after same salinomycin stimulation (PI positive and Annexin V negative, which was largely inhibited by necrosis inhibitor Nec-1 (Fig. 2g). More importantly, combination of Nec-1 and zVADfmk almost completely blocked salinomycin-induced cytotoxicity in U87MG cell (Fig. 2h). Together, these results suggest that salinomycin induces some apoptosis, but mainly necrosis in cultured glioma cells.

### Cyp-D is required for salinomycin-induced necrosis but not apoptosis in cultured glioma cells

**Fig. 3:**
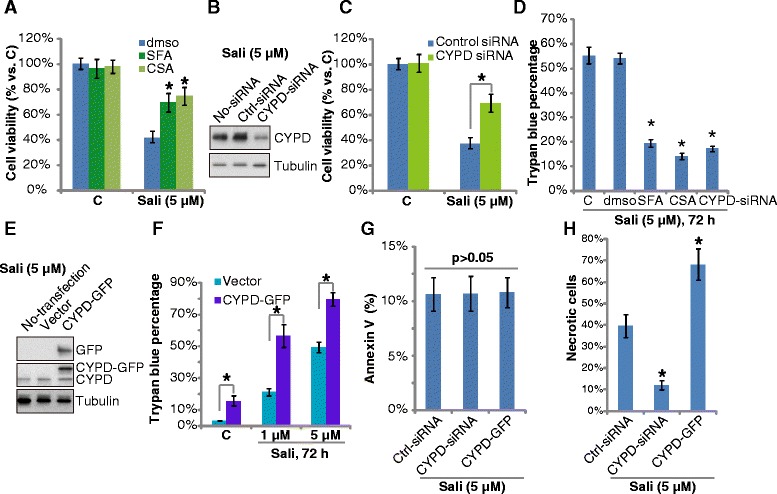
Cyp-D is required for salinomycin-induced necrosis but not apoptosis in cultured glioma cells. U87MG cells were pre-treated with the Cyp-D inhibitor CsA (0.1 μM) or SfA (10 μM) for 1 h, followed by salinomycin (Sali, 5 μM) stimulation, cells were further cultured, MTT assay (**a**, 72 h) and trypan blue staining (**d**, 72 h) were performed. U87MG cells transfected with scramble or Cyp-D siRNA (100 nM each, 48 h) were treated with salinomycin (Sali, 5 μM) for 72 h, cell viability and death were tested (**c** and **d**), expression of Cyp-D and tubulin was shown (**b**). Stable U87MG cells with empty vector (p-Super) or Cyp-D-GFP (1 μg/ml each) were treated with salinomycin (Sali, 1 or 5 μM) for 72 h, Cyp-D/tubulin expression (**e**) and cell death (**f**) were tested. Non-transfected control U87MG cells, Cyp-D siRNA-transfected U87MG cells or Cyp-D-over-expressing U87MG cells were treated with salinomycin (Sali, 5 μM) for 72 h; cell apoptosis (**g**) and necrosis (**h**) were tested by FACS. Experiments were repeated three times in this figure, and similar results were obtained. Error bars indicate SD. *p < 0.05 vs. dmso group (**a** and **d**). *p < 0.05 (**c** and **f**)

A number of recent studies have shown that the mitochondrial protein Cyp-D is required for stresses-induced necrotic, but not apoptotic cell death [16–19, 23, 31]. Thus, we tested the possible involvement of Cyp-D in salinomycin’s cytotoxicity in glioma cells. Results demonstrated that two Cyp-D inhibitors, sanglifehrin A (SfA) [32] and cyclosporin A (CSA) [23, 31], significantly inhibited salinomycin-induced viability reduction in both U87MG cells (Fig. 3a) and in U251MG cells (Additional file 1: Figure S2A). Further, siRNA-depleting Cyp-D (Fig. 3b) also alleviated salinomycin’s cytotoxicity in U87MG cells (Fig. 3c). Meanwhile, salinomycin-induced U87MG cell death was also inhibited by SfA, CsA or Cyp-D depletion (Fig. 3d). These results suggest that Cyp-D is important for salinomycin-induced cell death of glioma cells. To further support our hypothesis, we tested salinomycin’s effect in Cyp-D over-expressing glioma cells. As shown in Fig. 3e, we exogenously introduced wt-Cyp-D in U87MG cells. These cells were hyper-sensitive to salinomycin, as more cell death were achieved in Cyp-D over-expressing cells (Fig. 3f). We also noticed some spontaneous cell death in Cyp-D over-expressing cells (Fig. 3f). Significantly, salinomycin-induced cell apoptosis, shown by the Annexin V percentage, was not affected by Cyp-D depletion or Cyp-D over-expression (Fig. 3g), while cell necrosis induced by salinomycin was inhibited by Cyp-D siRNA, but was aggravated by Cyp-D over-expression (Fig. 3h). Together, these results indicated that the mitochondrial protein Cyp-D is required for salinomycin induced necrosis in cultured glioma cells.

### Salinomycin induces Cyp-D and p53 interaction in mitochondria

**Fig. 4:**
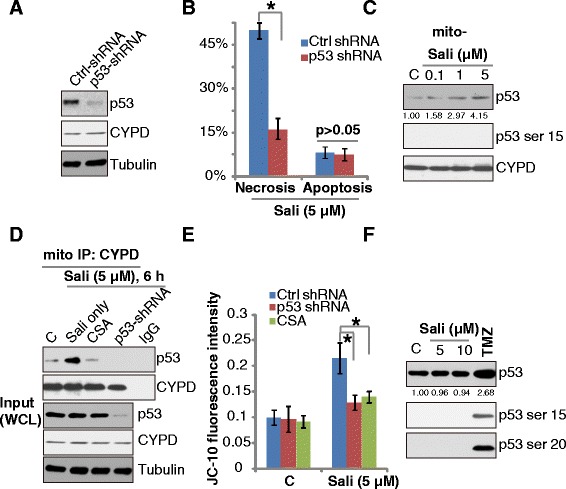
Salinomycin induces Cyp-D and p53 interaction in mitochondria. Expression of p53, Cyp-D and tubulin expression in stable U87MG cells with scramble-shRNA or p53-shRNA was shown (**a**). Above cell lines were treated with salinomycin (Sali, 5 μM) for 72 h, cell necrosis and apoptosis were tested by FACS (**b**). Cultured U87MG glioma cells were treated with indicated concentration of salinomycin (Sali) for 6 h, expression of p53, p-p53 (Ser 15) and Cyp-D in the mitochondrial fraction was tested by Western blots (**c**). Scramble- or p53- sh-RNA infected U87MG cells, pre-incubated with CsA (0.1 μM, 1 h), were treated with salinomycin (Sali, 5 μM) for 6 h, mitochondrial association of p53 and Cyp-D was tested (**d**), expression of above protein in whole cell lysates (WCL) was shown in input (**d**), mitochondrial membrane potential was also tested by JC-10 fluorescence assay (**e**). U87MG glioma cells were treated with salinomycin (Sali, 5/10 μM), or TMZ (5 mM) for 6 h, p-p53 and regular p53 were tested by Western blots (**f**). Error bars indicate SD. Experiments were repeated three times in this figure, and similar results were obtained. *p < 0.05 (**b** and **e**)

Previous studies have shown that Cyp-D-mediated necrotic cell death is through binding with p53 in mitochondria [19, 27, 33]. Since we have shown that Cyp-D is required for salinomycin’s cytotoxicity, we thus tested the role of p53 in the process. As demonstrated, p53-shRNA stable knockdown (Fig. 4a) significantly inhibited salinomycin-induced U87MG cell necrosis, while apoptosis was not affected (Fig. 4b). We noticed p53 mitochondrial translocation in salinomycin-treated U87MG cells (Fig. 4c), which formed a complex with the local protein Cyp-D (Fig. 4d). Similar results were obtained in U251MG cells (Additional file 1: Figure S2B). The mitochondrial complexation between Cyp-D and p53 was blocked by Cyp-D inhibitor CsA or p53-shRNA depletion (Fig. 4d). Our data suggested that the complexation was required for mPTP opening, as both CsA and p53 shRNA largely inhibited salinomycin-induced MMP loss (Fig. 4e). We failed to see p53 phosphorylation at Ser 15 or Ser 20, nor p53 upregulation by salinomycin, while temozolomide (TMZ) induced p53 phosphorylation and upregulation (Fig. 4c and f). Based on these data, we suggest that salinomycin induces p53 mitochondrial translocation to interact with Cyp-D, which is required for mPTP opening and programmed necrosis.

### ROS is required for salinomycin-induced necrosis in cultured glioma cells

**Fig. 5:**
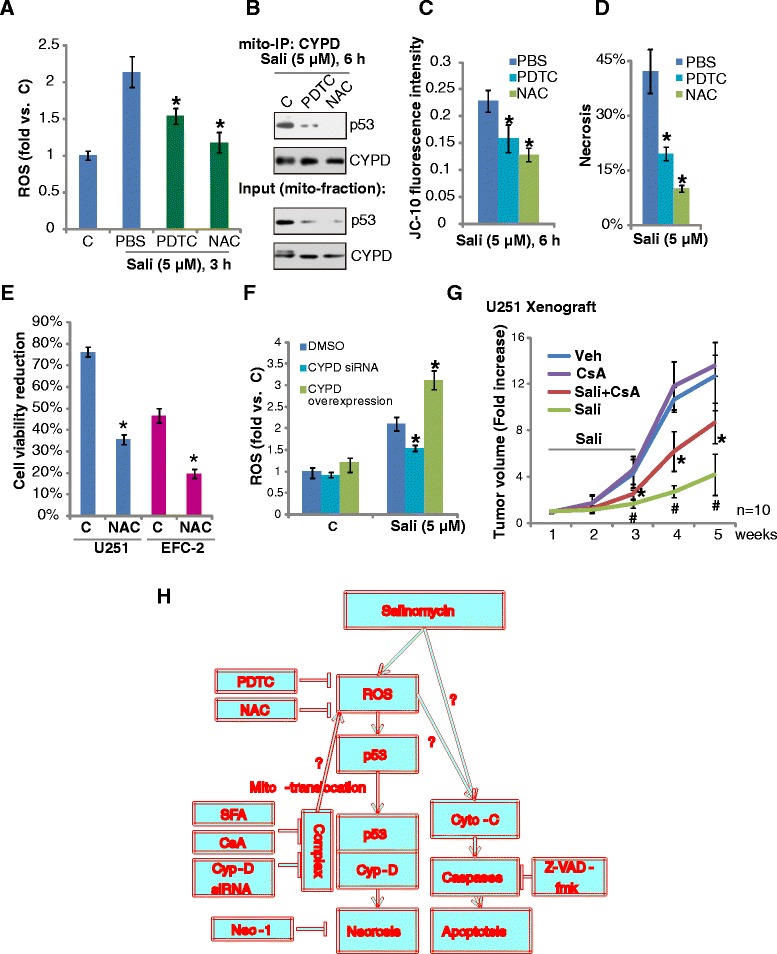
ROS is required for salinomycin-induced necrosis in cultured glioma cells. U87MG glioma cells were pre-treated with anti-oxidants NAC (50 μM) or PDTC (25 μM) for 1 h, followed by salinomycin (Sali, 5 μM) stimulation, ROS production was analyzed 3 h after stimulation (**a**), mitochondrial expression and association of p53 and Cyp-D were tested at 6 h after stimulation (**b**), JC-10 green fluorescence intensity was examined after 6 h (**c**); cell necrosis was analyzed similarly 72 h after salinomycin stimulation (**d**). The effect of NAC (50 μM, 1 h pretreatment) on salinomycin (Sali, 5 μM)-induced viability loss was tested by MTT assay in U251MG and EFC-2 cells (**e**). Non-transfected control U87MG cells, Cyp-D siRNA-transfected U87MG cells or Cyp-D-over-expressing U87MG cells were treated with salinomycin (Sali, 5 μM) for 3 h, ROS production was analyzed (**f**). (**g**) U251 cells (1 × 10^6^) were injected subcutaneously into nude mice as described in Materials and Methods, and treatment was started when the tumors reached 200 mm^3^. Salinomycin (5.0 mg/kg) and/or CsA (5.0 mg/kg) were administered intraperitoneally once daily for two weeks. Control mice received vehicle only, according to the same schedule. Tumor volume was measured by caliper with the formula: π/6 × width ^2^× length. *N* = 10 for each group. (**h**) The proposed signaling pathway of this study. Experiments were repeated three times in this figure, and similar results were obtained. Error bars indicate SD. * *p* < 0.05 vs. Sali only group (**a**, **b**, **d**, **f** and **g**). ^**#**^ *p* < 0.05 vs. vehicle group (**g**)

Next we tested the possible upstream signal for salinomycin-induced mPTP opening and necrosis. FACS results in Fig. 5a showed ROS production by salinomycin in U87MG cells, which are consistent with other studies [12, 34, 35]. We found that salinomycin decreased the activity of SOD in glioma cells, which could be one reason for ROS increase (Additional file 2: Figure S1). N-acetyl cysteine (NAC) and pyrrolidinedithiocarbamate (PDTC), two well known anti-oxidants inhibited ROS production by salinomycin. Significantly, both NAC and PDTC suppressed p53 mitochondrial translocation (Fig. 5b, input) and following Cyp-D association (Fig. 5b). Further, salinomycin-induced MMP loss, an indicator of mPTP opening, was also inhibited by these two anti-oxidants (Fig. 5c). These results indicate that salinomycin-induced p53 mitochondrial translocation, p53-Cyp-D complexation, and the subsequent mPTP opening might be dependent on ROS production. Based on these results, we predicted that ROS might also be important for salinomycin-induced necrosis. As a matter of fact, salinomycin-induced U87MG cell necrosis was alleviated by NAC or PDTC (Fig. 5d), which could explain that NAC inhibited salinomycin-induced viability loss in both U251 MG cells and EFC-2 cells (Fig. 5e). Interestingly, using the cells above, ROS results in Fig. 5f showed that salinomycin-induced ROS was inhibited by Cyp-D depletion, but was aggravated by Cyp-D over-expression. Thus, salinomycin-induced mPTP opening might play a vital role in ROS regulation in glioma cells. To evaluate the anti-tumor activity of salinomycin *in vivo*, we generated subcutaneous xenografts by inoculating the U251 cells to nude mice. Treatment with salinomycin elicited a marked inhibitory effect on U251 xenograft development (Fig. 5g). Such an effect by salinomycin was significantly inhibited by co-administration with CsA (Fig. 5g). Note that CsA alone failed to affect U251 growth *in vivo* (Fig. 5g). Further, the mice body weight was not significantly affected by salinomycin or with CsA, indicating the relative safety of this regimen (Additional file 3: Figure S3).

## Discussions

A recent study has demonstrated salinomycin to selectively kill breast cancer stem cells from tumorspheres and to inhibit tumor growth in mice [8]. Salinomycin has been investigated as a possible novel anti-cancer agent [8, 9]. In the current study, we found that salinomycin (0.1–10 μM) decreased the viability of cultured glioma cells in a concentration- and time-dependent manner. These results are consistent with previously published results showing that low concentrations of salinomycin inhibit the survival of many cultured cancer cells [9, 11, 34, 36–41]. Interestingly, we found that both apoptosis and necrosis were induced by cytotoxic salinomycin in cultured glioma cells, and necrosis appeared more important than apoptosis in contributing salinomycin’s cytotoxicity.

In the last decades after the discovery of p53, it has become increasingly clear that this protein plays a vital role in tumor suppression. Previously, it was thought that the tumor suppressive functions lied solely in p53-mediated apoptosis, cell cycle arrest, and senescence. However, more recent research has shown that anti-oncogenic activity of p53 can still occur in the absence of these downstream functions. More specifically, it was recently found that p53 is also a critical player for cell necrosis. Upon oxidative stress, p53 triggers mPTP opening by engaging in a physical interaction with Cyp-D [19], thereby inducing necrotic cell death in mouse and human cells. Cells/mice deficiency of Cyp-D or p53 were protected from necrosis induced by a various stimuli (i.e. hypoxia, calcium overload, and ROS). More recent studies have demonstrated that this Cyp-D/p53 association is also a key player for cancer cell necrosis induced by some anti-cancer drugs (cisplatin and doxorubicin) [26, 33], as well as anti-cancer herb medicine (i.e. curcumin) [42]. In the current study, we found that Cyp-D-p53 is required for salinomycin-induced necrosis in cultured glioma cells.

Here, we presented data showing that blockade of Cyp-D by siRNA-mediated depletion or pharmacological inhibitors (CsA and SfA) significantly suppressed salinomycin-induced MMP decrease and glioma cell necrosis. Further, the *in vivo* anti-tumor activity by salinomycin was also inhibited by CsA. These results suggest that an inhibitory effect of Cyp-D deficiency on salinomycin-induced cytotoxicity. On the other hand, Cyp-D overexpression facilitated salinomycin-induced glioma cell death. Thus, we proposed that Cyp-D dependent mPTP opening should play a vital role in salinomycin-induced cytotoxicity in glioma cells (Fig. 5h).

In the current study, we found that salinomycin induced p53 mitochondrial translocation without inducing its phosphorylation (Ser 15 and Ser 20). On the other hand, TMZ failed to induce p53 mitochondrial translocation or Cyp-D association (Data not shown), although it did promote p53 Ser 15 and Ser 20 phosphorylation. Thus, it is suggested that p53 Ser 15 and Ser 20 phosphorylations are not required for its mitochondrial translocation or Cyp-D complexation by salinomycin. It is possible, though, that other sites of p53 besides Ser 15 and Ser 20 could be phosphorylated by salinomycin, which might dictate its mitochondrial translocation. Salinomycin might also activate p53 in a phosphorylation-independent manner in glioma cells.

Another major consequence of mPTP formation is increased ROS production/accumulation, leading to release of oxidative stress from mitochondrial to cytosol. In the current study, we found that ROS was induced by salinomycin in glioma cells, which are consistent with previous findings in other cell lines [12, 34, 35, 41, 43]. Meanwhile, ROS appeared important for regulating mPTP opening through activating of p53, as NAC and PDTC, two well-known anti-oxidants, inhibited salinomycin-induced p53 mitochondrial translocation/Cyp-D association, mPTP opening and glioma cell death. Thus, ROS is the trigger for the salinomycin-induced p53-Cyp-D dependent cell necrosis. Interestingly, blockade of Cyp-D by siRNA-mediated depletion inhibited salinomycin-induced ROS production, while Cyp-D over-expressing cells showed a higher level of ROS with salinomycin stimulation. Thus, we propose the following model: in salinomycin-treated glioma cells, ROS production triggers p53-Cyp-D mitochondrial association to open mPTP, leading to secondary ROS production/accumulation in mitochondria and release from mitochondrial to cytosol, causing further oxidative damages, and cell necrosis (Fig. 5h).

## Conclusions

In conclusion, we found that ROS-p53-Cyp-D dependent programmed necrosis plays a major role in contributing salinomycin’s cytotoxicity in cultured glioma cells.

## Additional files

Additional file 1: Figure S2. U251MG cells were pre-treated with the Cyp-D inhibitor CsA (0.1 μM) or SfA (10 μM) for 1 h, followed by salinomycin (Sali, 5 μM) stimulation for 72 h, cell viability was tested by MTT assay (A). U251MG cells were treated with salinomycin (Sali, 5 μM) for 6 h, mitochondrial association of p53 and Cyp-D was tested (B), expression of indicated proteins in whole cell lysates (WCL) was shown (B). Experiments were repeated four times in this figure, and similar results were obtained. Error bars indicate SD. **p* < 0.05. Additional file 2: Figure S1. U87MG glioma cells were treated with indicated concentration of salinomycin (Sali) for 3 h, SOD activity was measured. Experiments were repeated four times in this figure, and similar results were obtained. Error bars indicate SD. **p* < 0.05 vs. untreated control (“C”) group. Additional file 3: Figure S3. Weekly body weights of nude mice with applied treatments described in Fig. 5. Error bars indicate SD.

## Abbreviations

CSA: Cyclosporin A
Cyp-D: Cyclophilin-D
mPTP: Mitochondrial permeability transition pore
MMP: Mitochondrial membrane potential
NAC: n-acetylcysteine
Nec-1: Necrostatin-1
PI: Propidium iodide
PDTC: Pyrrolidinedithiocarbamate
ROS: Reactive oxygen species
SFA: Sanglifehrin A
SOD: Superoxide dismutase
TMZ: Temozolomide
